# Short-term assist devices in postcardiotomy cardiogenic shock

**DOI:** 10.1186/s13054-019-2471-0

**Published:** 2019-06-03

**Authors:** Hasan Iner, Nihan Karakas Yesilkaya, Yuksel Besir, Gamze Gokalp, Orhan Gokalp, Levent Yilik, Ali Gurbuz

**Affiliations:** 1 Department of Cardiovascular Surgery, Izmir Katip Celebi University Ataturk Training and Research Hospital, Maliyeciler District 52, 172 St. No:1-4, 35550 Izmir, Turkey; 2 Department of Cardiovascular Surgery, Tunceli State Hospital, Tunceli, Turkey; 3 Department of Cardiovascular Surgery, Izmir Katip Celebi University, Faculty of Medicine, Izmir, Turkey; 4 Pediatric Emergency Department, Izmir Tepecik Training and Research Hospital, İzmir, Turkey

We congratulate Wang and colleagues for their study and very successful results [1]. Although the used short-term circulatory support system was venoarterial extracorporeal membrane oxygenation (VA-ECMO), the weaning rate reported by Wang et al., 64%, is quite high. Especially in postcardiotomy cardiogenic shock, whether or not cardiopulmonary bypass is used, frequently emerging situation is left ventricular dysfunction. Naturally, it is actually a short-term left ventricular support system (LVAD) that should be used for recovery of left ventricular functions. However, VA-ECMO is often preferred because of the ease of percutaneous placement in daily surgical practice, as in the study of Wang et al. But in our opinion, this is not a foolproof practice. Because VA-ECMO does not vent the left ventricle, so it cannot be expected to recover the post-cardiotomy left ventricle dysfunction. In particular, VA-ECMO, which is placed through the femoral artery, increases the afterload, makes the ventricle recovery even more difficult. If Wang and colleagues had used a real short-term LVAD (such as the Levitronix CentriMag) in all these patients, could weaning results have been much better? Another issue we wondered is the effect of ECMO duration on weaning or mortality rates. Because in some studies, as the duration of ECMO increases, weaning and survival rates increase [2] whereas in some other studies, an opposite result is mentioned [3]. In the study of Wang et al., patients who underwent ECMO less than 3 days had less mortality compared to those who underwent ECMO for 3–6 days. What are the comments of the authors on this subject by considering their own results?
