# Supplementary material for: Short-term assist devices in postcardiotomy cardiogenic shock
Source: Crit Care. 2019 Jun 3;23:199. doi: 10.1186/s13054-019-2471-0 (PMC6547548; doi:10.1186/s13054-019-2471-0)
Supplement: Related texts

# Reply: Authors’ response

**Authors:** Liangshan Wang, Feng Yang, Hong Wang, Xiaotong Hou

We are grateful to Iner et al. for their interesting and valuable comments on our paper.

Peripheral venoarterial extracorporeal membrane oxygenation (VA-ECMO) increases the left ventricle (LV) afterload, and LV overload increases wall stress and myocardial oxygen consumption, jeopardising LV recovery in postcardiotomy cardiogenic shock patients [4]. In our study, more than 80% of patients received VA-ECMO combined intra-aortic balloon pump, which might reduce LV afterload and increase coronary blood flow. Since left ventricular assist devices were not registered in China, no patients underwent ventricular assist device after VA-ECMO. The usefulness of VA-ECMO for these patients might have therefore been underestimated. As for the effect of ECMO duration on weaning or mortality rates, patients who underwent ECMO for 3–6 days had significantly lower mortality than those who used ECMO for < 3 days, which was similar with the results of the extracorporeal Life Support Organization (ELSO) registry [5]. Most of the patients who used ECMO for < 3 days could not be weaned from ECMO, which might account for our findings. The reasons for discontinuation in the early period included haemorrhage, organ failure and family request. Our study did not suggest that weaning should occur on a particular day in order to maximise survival. The duration of VA-ECMO depends on the underlying disease process.
